# How COVID-19 has exposed inequalities in the UK food system: The case of UK food and poverty

**DOI:** 10.35241/emeraldopenres.13539.2

**Published:** 2020-05-13

**Authors:** Madeleine Power, Bob Doherty, Katie Pybus, Kate Pickett

**Affiliations:** 1 Health Sciences, University of York, York, Yorkshire, YO10 5GD, UK; 2 The York Management School, University of York, York, Yorkshire, YO10 5GD, UK

**Keywords:** food insecurity, food banks, food supply, inequality, poverty, Covid-19, coronavirus, pandemic

## Abstract

This article draws upon our perspective as academic-practitioners working in the fields of food insecurity, food systems, and inequality to comment, in the early stages of the pandemic and associated lockdown, on the empirical and ethical implications of COVID-19 for socio-economic inequalities in access to food in the UK. The COVID-19 pandemic has sharpened the profound insecurity of large segments of the UK population, an insecurity itself the product of a decade of ‘austerity’ policies. Increased unemployment, reduced hours, and enforced self-isolation for multiple vulnerable groups is likely to lead to an increase in UK food insecurity, exacerbating diet-related health inequalities. The social and economic crisis associated with the pandemic has exposed the fragility of the system of food charity which, at present, is a key response to growing poverty. A vulnerable food system, with just-in-time supply chains, has been challenged by stockpiling. Resultant food supply issues at food banks, alongside rapidly increasing demand and reduced volunteer numbers, has undermined many food charities, especially independent food banks. In the light of this analysis, we make a series of recommendations. We call for an immediate end to the five week wait for Universal Credit and cash grants for low income households. We ask central and local government to recognise that many food aid providers are already at capacity and unable to adopt additional responsibilities. The government’s - significant - response to the economic crisis associated with COVID-19 has underscored a key principle: it is the government’s responsibility to protect population health, to guarantee household incomes, and to safeguard the economy. Millions of households were in poverty before the pandemic, and millions more will be so unless the government continues to protect household incomes through policy change.

## Background: A fragile just-in-time food system and rising food insecurity

The COVID-19 pandemic, and associated pressures on food access, has revealed stark inequalities in the UK system of food supply and food distribution. During extreme scenarios such as this, consumers sometimes demonstrate behaviours designed to mitigate against the risk of not being able to purchase food, or indeed other items, at a later date ( Slonim & Garbarino, 2009; Sterman & Dogan, 2015) - for example, since the beginning of March 2020 we have seen evidence of stockpiling, hoarding, and panic buying. Stockpiling is an accumulation of goods predominantly motivated by a desire to minimise the loss of, or the risk of losing access to, certain products, and may arise due to a belief that a product is in short supply, will soon no longer be available, or that its price is likely to increase. In the UK, this has led to panic buying and empty shelves in supermarkets, jeopardising the food security of more vulnerable groups. According to the British Retail Consortium (BRC), the demand spike in March 2020 exceeded that forecast for peak periods, such as Christmas ( BRC, 2020a). Lockdown has led to 503 million more (+38%) in-home meals eaten per week creating further pressures on food supplies ( AHDB, 2020). Supermarkets have responded to this increased demand via a series of strategies: recruiting extra staff, particularly through deployment from other sectors; introducing limits on purchase volumes; and scaling-up online deliveries.

The situation has exposed vulnerabilities embedded within the UK food system, including dependence on just-in-time (JIT) supply chains, which operate to source produce at competitive prices from overseas territories in an efficient manner. It is widely known that the UK imports 48% of its food - in fact, for fresh fruit it is 84% and for vegetables 46% ( ONS, 2018). It is also important to note that the UK is heavily reliant upon European Union (EU) countries, such as Spain for vegetables and salads and Italy for ambient goods. However, despite advances in supply chain technology and logistics, this increased sourcing efficiency has paradoxically made supply chains more vulnerable to disruptions ( Lang, 2020). Approaches including ‘lean sourcing’, JIT systems, standardised components, and reductions in the supply base have tended to neglect the systemic risks caused by exogenous shocks or disruptions to the supply chain ( Christopher & Peck, 2004; Lee & Rammohan, 2017). Writing in mid-late March 2020, it appears that COVID-19 is testing food supply chains to their limits and yet, according to the UK Government, we are in the early stages of the pandemic, in terms of its direct and indirect health, economic and social impacts.

Heightened vulnerabilities within our food system, exposed by the economic and social crisis associated with the COVID-19 pandemic, occur against a backdrop of growing inequalities in access to food in the UK, themselves exacerbated - caused - by ‘austerity’ policies since 2010 ( Lamie-Mumford & Green, 2017). These inequalities divide households and individuals who have adequate income, mobility and social support to be able to access food through ‘normal’ channels - purchasing fresh or prepared produce at shops, cafes and restaurants - from those who do not ( Garthwaite, 2016; Lambie-Mumford, 2017; Loopstra **et al.**, 2015). The salience of food inequalities prior to COVID-19 and, especially, the widespread use of food banks, can largely be attributed to the £30 billion of cuts in working-age social security initiated by the Coalition government in 2010. Between April 2018 and March 2019, the Trussell Trust’s network of food banks distributed 1.6 million food parcels, a 26-fold increase on 2010 ( Trussell Trust, 2019a). Trussell Trust food banks represent approximately two thirds (1200 in total) of all food banks operating in the UK - there are over 800 food parcel distribution projects or food banks operating outside of the Trussell Trust’s network of food banks ( Independent Food Aid Network, 2020) - and, hence, their statistics underestimate food bank use. For instance, across Scotland, between April 2017 and September 2018, 221,977 food parcels were distributed by 84 independent food banks, roughly equivalent to the 258,606 food parcels distributed by Trussell Trust food banks ( Goodwin, 2018).

The number of households who have ‘limited access to food’ due to ‘lack of money or financial resources’ (food insecurity) ( FAO, 2017) is poorly reflected in statistics on food bank use. The Food Standards Agency ( FSA, 2017) estimates that 13% of UK adults are ‘marginally food insecure’ and 8% are moderately or severely food insecure. The variation between the extent of food bank use and the prevalence of food insecurity can be sharply captured at the local level: in York, for example, a survey of households with children found that only 20% of those in the sample reporting food insecurity had ever used a food bank ( Power, 2019). Single individuals, particularly men, are a key demographic of food bank users ( Sosenko *et al.*, 2019) and, therefore, it is possible that this survey underestimates the extent of the discrepancy between food insecurity and food bank access. Nevertheless, the variation between the extent of food banks use and the numbers experiencing food insecurity is also reflected in the work of Macleod *et al.* (2019) in Glasgow (just over one-in-six of those who had experienced difficulty paying for food had used a food bank) and Loopstra *et al.* (2019a). In the latter study, the estimated number of adults using Trussell Trust food banks in 2016–17 was 321,599, contrasting sharply with the estimated number of food insecure - 10.2 million - based on the FSA data.

Poor diet and broader experiences of food insecurity are important factors in lower life-expectancy, weakened immunity ( Health Foundation, 2020), and poorer mental health and wellbeing ( Tarasuk *et al.*, 2013). Food insecurity has long-term and life-changing consequences: it is highly associated with both malnutrition and obesity amongst adults and children ( Martin & Ferris, 2007); it compromises educational outcomes amongst children ( Jyoti *et al.*, 2005); and hinders stable employment amongst adults ( Lent *et al.*, 2009).

This article draws upon our perspective as academic-practitioners working in the field of food insecurity, food systems, and inequality to comment on the empirical and ethical implications of COVID-19 for socio-economic inequalities in access to food in the UK. Alongside roles as academics, Doherty and Pybus have policy experience; Power co-chairs a national network of food aid providers; and Pickett and Power have experience working within charities. Pickett also has over twenty years’ of experience working in the field of inequalities. In the context of existing literature on food insecurity, food systems, and socio-economic inequalities, as well as our own body of research on inequalities in food access, we chronicle and assess the multiple inequalities exposed by COVID-19, in access to food; within the food aid sector; and between retailers and food aid providers.

This is an unprecedented health and economic crisis, with unpredictable and fast-moving events, accompanied by major policy changes. It is possible that the challenges, events, and policy changes documented in this article - written in mid-late March 2020 - will become out-dated, even in the short-term. The evidence drawn upon is, necessarily, fragmentary and fluid. In the light of the context in which we are writing, a majority of our evidence is derived from grey literature and media outlets; the absence of data of a complete and consistent quality challenges how we make policy recommendations to ameliorate the inequalities we identify. As a consequence, our conclusions and recommendations should be considered as a starting point for further debate, rather than a definitive answer to the complex empirical and ethical issues we discuss. Nevertheless, we believe that the process of chronicling and reflecting upon events as they happen during the period of the pandemic - within the confines of the available evidence and our own positionality - is itself important in understanding how we, as a society, respond to major challenges and in considering how we can prevent the escalation of existing inequalities that may result from COVID-19.

It is important to make clear that at no point are we under-estimating the tireless efforts of staff and volunteers in food banks and other charitable food aid providers in supporting those who lived in poverty before this crisis and those who will be pushed into poverty as a result of it. This paper is a consideration of inequality and poverty, and the charitable organisations that respond to it, in the context of wider structures and broader processes.

The paper is structured as follows. To begin, we consider the inequalities in income, diet, and access to food in the UK exposed and exacerbated by the social and economic crisis associated with COVID-19. This is followed by an assessment of the resilience - and the uniformity of this resilience - of the UK food banking system in the context of the pandemic. We consider the extent to which COVID-19 has changed the relationship between supermarkets and food aid providers, and reflect upon political and ethical debates surrounding food charity in the UK that have been further illuminated by the pandemic. To conclude, we make a series of recommendations for social security policy, ‘emergency’ food provision, and food retail, and consider how the COVID-19 pandemic may and could change socio-economic inequalities in the UK.

## Commentary: multiple food inequalities in the context of COVID-19

### Inequalities in income, diet, and access to food

The social and economic fallout of COVID-19 has exposed the extreme precarity of large segments of the UK population. Reports from food aid governance organisations and published on media platforms suggest that food banks have seen sharp increases in demand for food due to sudden unemployment and reduced wages as organisations implement redundancies and scale down staffing ( Cipriani, 2020). Panic buying, and consequent food shortages on supermarket shelves, has jeopardised the food security of low income households and individuals in vulnerable groups. Many low income households are financially unable to stockpile and, confronted with only the most expensive versions of products, cannot purchase adequate food. Our research on the lived experience of food on a low income shows the complicated food management and shopping strategies adopted by low income households to maintain food security, including buying reduced cost items and cheaper, own-brand products; shopping at multiple supermarkets to find the cheapest items; and budgeting on a long-term basis ( Power *et al.*, 2018b). Insecure employment and poor availability of food in supermarkets may render such strategies impossible, forcing households to access food from food banks or go without food entirely.

Early quantitative evidence shows the dramatic effects of COVID-19 on household income and food security. Survey data collected by YouGov for the Food Foundation finds that 2% of respondents, equivalent to more than one million people, have lost all of their income, whilst 6% have had to borrow money or take out personal loans as a result of COVID-19. Since the lockdown came into effect in early March 2020, 3% of respondents have gone a whole day without eating; 14% report that someone in their household has had to reduce or skip meals because they could not access or afford sufficient sustenance (The Food Foundation, 2020a). This data suggests that adults with young children - a population historically under-represented at food banks - are amongst the groups most affected by COVID-19.

The acceleration of COVID-19 has led to rapid changes in government policy, as employers across multiple sectors close premises to help slow the spread of the virus. The extent to which the policy response to the economic and social fallout of COVID-19 is sufficient to prevent increases in poverty and food insecurity remains, at the time of writing (March 2020), highly questionable. Protections for some businesses and employees have now been outlined, but a delay between the announcement of social distancing measures, leading to closures, and the implementation of employee protection schemes has already precipitated redundancies. In addition, some companies have gone into administration whilst others, who do not qualify for Government grants, are at continued risk of doing so, or face mounting debts with potential consequences for jobs ( Financial Times, 2020). Employees have, however, been offered a number of protections that may help to militate against loss of income ( Department for Business, Energy and Industrial Strategy, 2020). These include being able to claim statutory sick pay from the first day of illness or self-isolation; and the introduction of the Coronavirus Job Retention Scheme (CJRS), in which 80% of salary costs will be paid by the Government up to £2,500 per month pre-tax, with an equivalent scheme for those who are self-employed. In addition to mortgage holidays and new rules on evictions in force for the next three months, these measures *may* mitigate large scale income shocks amongst the majority of the population, so limiting the extent of increases in food insecurity.

Less clear, however, is the level of protection the measures will provide in the short- to medium- term. Employers, for example, are not required to fund the difference between salary costs paid by the Government under the retention scheme and the usual salary of the person ( Department for Business, Energy and Industrial Strategy, 2020). This means that some households could see their income reduce by a fifth for a sustained period of time, during which costs for utilities and food may increase as a result of families staying at home. In addition, those on zero hours contracts are not automatically entitled to be part of the CJRS and their continued receipt of wages is at the discretion of employers. Reduced incomes may be exacerbated by existing financial difficulties, for example, the Office for National Statistics (2018) estimates that two thirds of the poorest 40% of UK households have financial debts (excluding those relating to property). Reflecting such economic precarity, almost half of UK adults (44.3%), surveyed since the onset of COVID-19 in the UK, stated that they expect their financial position to worsen over the next year ( Office for National Statistics, 2020).

Further support is being offered through the social security system. New and existing Universal Credit and Working Tax Credit claimants will see an increase in payments of £20 per week, and an assumed level of earnings used to calculate entitlement (the minimum income floor) has been removed for the self-employed ( Department for Work and Pensions, 2020). Housing benefit will increase for all new and existing claimants to cover up to the 30th percentile of market rents ( Department for Work and Pensions, 2020).

However, important issues already known to increase poverty amongst social security claimants remain unaddressed. Universal Credit continues to be contingent on overall household income, whilst the benefit cap and the two-child limit (restricting the child allowance in Universal Credit and tax credits to the first two children in a family if the third and subsequent children were born after April 1 2017) remain in place, severely disadvantaging larger families. At the time of writing, there appear to be no plans to reduce the five-week wait for Universal Credit, a key driver of income shocks associated with food insecurity ( Trussell Trust, 2019b). Job centre closures and increased application demand (950,000 new applications in the last two weeks of March 2020) means that the majority of new claimants are being directed to complete their Universal Credit application online; however, reports suggest significant queues of over 100,000 applicants to complete online identity verification processes ( BBC, 2020b; Proctor, 2020). This is leading to delayed claims, and it is likely that the situation will worsen as more people apply over the coming weeks. A planned increase in the Department for Work and Pensions workforce ( Department for Work and Pensions, 2020) may assist in reducing some of the pressures but immediate responses are also needed to ensure people are able to access social security support as quickly as possible. It is impossible to comment with certainty on the impact of the continued five-week wait on poverty and food insecurity; however, based on the unprecedented increase in applications and previous evidence suggesting that around 60% of new Universal Credit claimants require advance payments ( Sharma, 2018), it is likely that large numbers of households will need to access this facility and subsequently receive lower monthly amounts than their initial entitlement, with longer term implications for household incomes.

The measures to support households in these unprecedented and challenging circumstances are welcome and - in the short-term - may assist in mitigating against a large-scale increase in poverty, including food poverty and insecurity. The longer-term implications are as yet uncertain. For the lowest income households, increases in Universal Credit, should these remain for the foreseeable future, may reduce risks of food insecurity. It is possible, however, that in the longer term, the pandemic could place higher numbers of households in precarious financial circumstances.

### Emerging fragilities within the UK food aid system

The social, economic and health crisis surrounding and stemming from COVID-19 has exposed the fragility of the system of ‘emergency’ food aid in the UK. Before the crisis, food banks (and other community food providers) largely received food in three ways: surplus food redistributed directly by supermarkets or indirectly by charities, such as FareShare; food donated by individuals either within the supermarket itself (for instance, in a donation basket), directly to the food bank, or through other charities/organisations (e.g. a local church); and food purchased in bulk by the food bank from local supermarkets and shops (see Table 1). The social and economic crisis surrounding COVID-19 has jeopardised these food supply chains. Individual food donations have dropped sharply as households prioritise their own food supply, whilst many food banks are unable to purchase the non-perishable items required for standard food parcels, due to supermarket rationing and the poor availability of much of this produce as a result of stockpiling ( Butler, 2020).

**Table 1.** Food supply systems to food banks.

Food supply	Partners involved	Challenges posed by Covid-19 pandemic
Surplus and non-surplus food direct from supermarkets (national-level)	Supermarkets, Fairshare, City Harvest, The Felix Project, The Bread and Butter Thing (TBBT), food banks, and other food aid providers	Dependent on partnerships established prior to Covid-19; immense logistical challenge of setting up new partnerships, food supplies, and deliveries in short time period; dependent on availability of supermarket produce in context of high demand from customers
Surplus food direct from shops (local-level)	Supermarkets, retailers, food banks, and other food aid providers	Lower availability of surplus food due to stockpiling; dependent on availability of volunteer and non-volunteer delivery staff; dependent on existing relationships, prior to Covid-19
Food donated by individuals in supermarkets	Food banks	Much reduced donations by individuals due to supermarket rationing and individual self-stockpiling
Food purchased in bulk from supermarkets/shops by food banks	Food banks	Supermarket rationing, and lower stock availability vastly limits food parcel items that can be purchased

Volunteers are integral to the provision of food charity in the UK. Between 2016 and 2017, volunteers contributed a total of 4,117,798 hours of labour supporting food banks, including distributing and caring for service users within the food bank, stock-taking, fundraising, collecting and delivering food, and inputting data ( IFAN, 2017). Before the pandemic, 75% of independent food banks relied on five or more volunteers each week ( Loopstra *et al.*, 2019b). These volunteers tend to be older (over 70); social distancing rules have caused many of these volunteers to stay away from food banks, placing considerable additional pressure on volunteers and staff continuing to distribute food.

Since 2010, there has been a rise in food aid responses that specifically target families with children, in recognition of additional food costs and a need to ensure that children do not experience hunger - for example, breakfast clubs and holiday hunger schemes ( Garnham, 2020). Whether this provision will prove adequate, or even viable, in the context of potentially increased child poverty is questionable. Indeed, and as noted above, emerging survey data suggests that adults with young children are particularly affected by COVID-19 ( The Food Foundation, 2020b). A combination of school closures, shortages and the compromised availability of support from emergency food provision creates a perfect storm for families on a low-income. Children who have key workers as parents or who are defined as vulnerable, for example, looked after children and those with a child protection plan or special educational needs, will be able to remain in school but most children will now be unable to attend ( Department for Education, 2020). Low income families with children who are eligible for free school meals will instead be provided with vouchers for local supermarkets, paid at a higher rate to reflect the increased costs attached to buying from supermarkets and the inability to purchase wholesale as schools do when providing these meals ( Department for Education, 2020). How far these vouchers enable families to purchase nutritional meals will depend on the availability of supermarket food and the impact of stockpiling. Whilst this response should ensure that these families are not disadvantaged in terms of diet, a recent poll finds that around 40% of adults with children have not yet received a substitute for free school meals ( The Food Foundation, 2020b). There are also questions to be raised around the adequacy of provision and lack of choice offered by the vouchers, as well as about the potentially stigmatising process of accessing a voucher in the first place.

### Changing relationships between retailers and food charity, and inequalities between food aid providers

The COVID-19 pandemic has changed and sharpened the relationship between retailers and food aid providers, and exposed the reality of food insecurity in the UK: it is primarily a question of the distribution of food supply, not necessarily food supply itself. Food worth £1billion that would be on supermarket shelves is now within households ( British Retail Consortium, 2020a), whilst food banks struggle to access sufficient food supplies and food insecurity looks set to increase. The high profile of the Trussell Trust as both a national organisation and a local food provider, coupled with food reserves and well-established links with supermarkets nationally and locally, has underpinned a dramatic increase in financial donations from retailers (and individuals) to the Trussell Trust and its network of food banks since the onset of COVID-19 in the UK. In conjunction with Fareshare, the Trussell Trust has received £5 million from their established charity partner, Asda ( Trussell Trust, 2020a), whilst the John Lewis Partnership, has pledged £75,000 to the Trussell Trust, in the first instance ( British Retail Consortium, 2020b). These initiatives will greatly enhance the resilience of the Trussell Trust during the pandemic. However, independent food banks have seen no equivalent size donations. Pressures on food supply, alongside increased demand and reduced volunteer numbers, has pushed some - especially independent - food banks to ‘breaking point’ and caused others to close entirely ( Bulman, 2020).

It may be too early to unpick the relative experiences of Trussell Trust and independent food banks during this period - indeed, there have been emerging reports of Trussell Trust food banks closing, suggesting similarities in experiences of the pandemic ( Wood, 2020), whilst Morrison’s provision of food worth £10 million to both independent and Trussell Trust food banks (announced in the week of 30 March 2020) points towards some parity ( BBC, 2020a). Nevertheless, long-established relationships with supermarkets, including Asda and Tesco, have supported Trussell Trust and FareShare food supply chains in times of crisis ( FareShare, 2020; Trussell Trust, 2020a). Independent food aid providers have historically resisted these relationships with large retailers, due to ethical concerns about the corporatisation of food aid ( Independent Food Aid Network, 2018), or have established relationships more informally and at a grass-roots level, rendering such supply chains vulnerable to disintegration.

### Political and ethical debates on food charity illuminated by COVID-19

The fragility of UK food charity, the extent of which has been exposed by the pandemic, alongside apparent inequality between food aid providers, has thrown into sharp relief the reality of food aid in the UK. Despite frequent discursive framing as an ‘emergency’ source of food, or ‘emergency’ service ( Wells & Caraher, 2014), many food banks have proved unreliable and fragile - in terms of volunteers, food supply, and premises - in the context of the health, economic and social crisis - the ‘emergency’ - of the COVID-19 pandemic. Whilst previous research (before the crisis) has documented elements of fragility in the food bank system - notably the potential inability of food charity to provide food or financial security due to its reliance on voluntary donations and voluntary labour (see Loopstra *et al.,* 2019c) - the current crisis has underscored the depth and scale of this fragility, as well as its detrimental implications for service users - the full extent of which is yet to be apparent. Food banks and food charities that have proved resilient have tended to be those with strong (or strengthening) corporate partnerships, raising questions about the importance of the corporatisation of food aid - itself highly contentious (see Fisher, 2017; Riches, 2018) - to the viability of food charity in the UK.

### Inclusivity, eligibility, and vulnerability

Referral systems, in which third-party agencies act as a ‘gateway’ to food banks by issuing a voucher that can be redeemed for a food parcel, have proved unviable in the context of increased demand, alongside the closure or partial shut down of many referral agents. The Trussell Trust broadly operates on a referral-only model ( Trussell Trust, 2020b), and 60% of independent food banks report requiring referrals from third-party agencies ( Loopstra *et al.*, 2019b). As ‘gateway’ agencies reduce face-to-face service provision or close down entirely, a voucher system may become untenable. An e-referral system (currently being developed as a partnership between Citizens Advice and the Trussell Trust), a possible short-term response, may compromise the inclusivity of food banks and could have long-term implications for the organisation and governance of the food bank sector. A voucher system could be particularly problematic for some individuals - for example, those who are older or who have disabilities, who may not have access to telephone or online communication methods if they need to request this support. Whilst a centralised referral system funded and overseen by the Trussell Trust could have long-term implications for the independence/autonomy of some ‘independent’ food banks, as well as the diversity of the food banking system.

There are important ethical questions about the distribution of food in the context of supply shortages and increased demand that are yet to be subject to public discussion and, related to this, how food banks are and *should be* responding to presently defined ‘vulnerable groups’. The government proposes two categories of vulnerability in the context of COVID-19, ‘extremely vulnerable’, those at ‘very high risk’ from COVID-19 due to health status and consequently required to ‘shield’, and those at high or increased risk from COVID-19 ( Public Health England, 2020). The latter ‘vulnerable’ category, which includes people over 70, regardless of whether they have an underlying health condition; people with an underlying health condition; and pregnant women, are required to self-isolate, but unlike the former, do not automatically receive assistance (for instance, food parcels delivered their home) from central government ( NHS, 2020). It is, therefore, possible that this group may now struggle to consistently and safely access nutritious food. This newly defined ‘highly vulnerable’ group is likely to be heterogeneous - many of its members may resist being categorised as vulnerable - and the extent to which its members will be ‘at very high risk’ from COVID-19 may be context specific and situational (see Jackson & Meah, 2017). Nevertheless, it is likely that, as a result of self-isolation and compromised food supply at the local level, members of this newly defined group may seek and/or become dependent upon support from local food charities, such as food banks.

It is unclear how food bank entitlement may be changed amidst COVID-19. In a context of limited food supply and reduced operational capacity, who should receive food - self-isolating individuals defined by government as ‘vulnerable’ or the ‘traditional’ food bank client: individuals and households who may be economically vulnerable - not a category proposed by government - as a result of low income or poverty? There is a growing body of literature on how food banks exacerbate and give material form to the discursive division of the ‘deserving’ and the ‘undeserving’ poor ( May *et al.*, 2019). The new heterogenous group of self-isolating vulnerable individuals - older adults, pregnant women, and those with pre-existing health conditions - who may be reliant on food banks in the immediate- and longer- term adds a new dimension to this debate. Are these individuals, whose need for food support is a product of demography and health status, more entitled to food than those whose use of the food bank is attributable to low/no income? Are these individuals, like those who used food banks before the crisis, also subject to a cap on the number of vouchers for food that can be received during a certain time period? Given the well-established link between poverty and poor mental and physical health ( Health Foundation, 2020), can a clear distinction be made between individuals seeking food support due to income and those requesting assistance due to health? Whilst a division between income and poor health will be clear-cut for some members of the government’s ‘highly vulnerable’ group, for others it may be more ambiguous and/or situational (see Jackson & Meah, 2017).

### Social distancing and stigma

Social distancing rules have modified interactions within some food banks: instead of being invited into a building, service users may be given a pre-packed food bank parcel, often at the threshold or delivered directly to their home ( Trussell Trust, 2020c). There is a danger that care, sign-posting, and choice - essential to the dignity and agency of service users - will be removed from the food bank interaction. This may undermine the role of food banks in addressing social isolation and acting to prevent the reasons for food bank attendance - the root causes of food insecurity - through onward referral, and may exacerbate the pre-existing stigma of food bank use. The additional ‘policing’ of the movement of people (where to stand, sit, hand over the voucher) may be ‘doubly stigmatising’. Such rules, if conveyed insensitively, are likely to intensify feelings of shame, particularly for people who are using the food bank for the first time. The pre-packaged parcel of non-perishable food may not only undermine the health of service users, exacerbating diet related inequalities, but also erode cultural inclusivity and further exclude ethnic minority groups from food banks. In doing so, it may increase income and health inequalities between ethnic groups (for an analysis of cultural exclusivity and food charity see Power *et al.*, 2018a), potentially exacerbating existing risk factors - notably race - for COVID-19 ( Pareek *et al.,* 2020). 

Nevertheless, the impact of social distancing rules on interactions within food banks may not be uniform, there may be ambiguity and heterogeneity to approaches by staff, volunteers, and service users. The Trussell Trust operates on a franchise model with each Trussell Trust food bank constituted as a separate charity or organisation, whilst the Independent Food Aid Network operates as a loose membership organisation providing guidance, rather than direction, to its members. In the absence of centralised instruction, there is likely to be considerable variation at the grassroots level. For instance, interactions within food banks may be contingent on layout and volunteers in each distribution centre. Attempts may be made to ensure dignity via the manner in which social distancing rules are communicated, by providing advice or signposting where possible, and in offering some choice in the pre-made food parcels. Food parcels, made by food bank volunteers, may also be distributed to social services and council hubs in order to bypass the need for vouchers and ease the physical and bureaucratic burden on individuals to travel to the food bank.

### Institutionalisation amidst government partnerships

The difficulties experienced by food banks in responding to rising destitution and food insecurity during this period are set against a backdrop of protracted austerity, a period in which the size of the welfare state and local authority capacity has diminished sharply whilst the number of food banks responding to rising destitution has proliferated ( Lambie-Mumford, 2017). Food banks have increasingly become embedded within local welfare systems, with local authorities and public health and social care professionals, such as GPs, acting as referral partners. The centrality of food banks and other food charities to central and local government response to the economic and social crisis affiliated with COVID-19 regnites (well-rehearsed) debate as to whether charitable food aid is - and can ever be - an acceptable and effective alternative to the state in responding to the basic needs of citizens (see, for example, Cloke *et al.*, 2017; Dowler & O’Connor, 2012; Lambie-Mumford & Dowler, 2014; Power *et al.*, 2017).

Government response to growing destitution and food insecurity, as well as to isolation, emanating from COVID-19 appears to be coloured by regional priorities and budgets. Notably the Scottish government’s response - a cash-first approach via an additional £45 million investment in the existing £35.5 million Scottish Welfare Fund, combined with additional financial and organisational support for food charities ( Scottish Government, 2020) - contrasts sharply with that of the English administration, which is yet to invest significantly in hardship funds or address sudden drops in household income, for instance as a consequence of the Universal Credit five week wait.

Many local authorities in England have collaborated with food aid providers at the local level to coordinate food distribution to both government-defined vulnerable groups and those who may be vulnerable as a result of income-level or income-shock, however the capacity of local authorities to respond to this crisis themselves, rather than relying on local charity partners, may be influenced by the uneven geography of budgetary cuts since 2010 ( Gray & Barford, 2018). Furthermore, the ability of Local Government Resilience Forums, pivotal to local crisis response, to coordinate essential food distribution at a local level may be additionally compromised by limited knowledge of the scale of food aid provision locally and nationally, and poor understanding of the number and demography of food insecure households before this crisis. Food charities, operating at capacity prior to COVID-19, may be poorly placed to assist such government-organised food distribution. Embedding partnerships between local authorities and food aid providers during the crisis, in the absence of effective financial support for low income households, risks further institutionalising food aid as part of a denuded welfare state.

### A future informed by mutuality and solidarity?

Community response to the social and economic fallout of the virus has been impressive and swift: thousands of ‘mutual aid’ groups have been organised across the UK, representing the pre-existence of mutuality at the local level and building further solidarity between community members. Similarly, emerging partnerships around food distribution within regions may be an illustration of rapid innovation around food supply, in addition to food distribution, at the local level - notably, initiatives (local and national) started from the out-of-home sector to help increase the supply of food to vulnerable groups. This has resulted in new alliances between local food charities and local restaurant chains, for example the London Food Alliance and Feed Britain, a new online national delivery service supplying fresh grocery produce and ready made meals to both NHS staff and vulnerable groups ( Catering Today, 2020; Independent, 2020). This may signal a more progressive food future, shaped by reciprocity and mutual aid. Further analysis as the crisis progresses will be required to ascertain the emancipatory potential of grassroots responses to COVID-19.

## Recommendations

The COVID-19 pandemic has revealed the profound insecurity of large segments of the UK population and exposed the fragility of the system of food charity that, at present, is a key response to such precarity. A vulnerable food system, with just-in-time supply chains, has been challenged by stockpiling. Resultant food supply issues at food banks, alongside rapidly increasing demand, has undermined many food charities, especially independent food banks. The food aid system appears to be unable to cope when faced with a health and economic emergency. In the light of this analysis, we make a series of recommendations for social security policy, ‘emergency’ food provision, and food retail. Developing policy recommendations in the context of an ‘emergency’ situation, amidst rapidly changing events, and with limited complete and high quality evidence presents considerable challenges. We recognise that our recommendations should be considered as a possible response, rather than a definitive solution, to existing and emerging inequalities. However, we argue that it is preferable to base policy changes on incomplete evidence, rather than abstain from providing policy recommendations at all, given the possible alternative of adverse effects on those already, and on the cusp of, experiencing poverty and hardship.

### Social security

It is evident that households require higher and more secure incomes to avoid destitution in the immediate response to the pandemic (within three months of March 2020); in the medium-term, whilst strict social distancing endures; and into the longer-term, during the aftermath of the pandemic. As discussed above, the five week wait for Universal Credit and associated advance payments may exacerbate poverty and food insecurity arising from/exacerbated by COVID-19. We therefore urge the Government to end the five week wait for Universal Credit, by providing grants, rather than advance payments which must be repaid, and streamline the processing time.

We echo the Joseph Rowntree Foundation’s (2020) call for further social security protections including suspending all benefit deductions for the next six months, increasing the child element of Universal Credit, and suspending the two child limit. In addition, we support the Child Poverty Action Group’s recommendation to add £10 per week to child benefits. This would reduce child poverty by up to 5% and have a greater impact than the recently announced increases to Universal Credit and tax credits ( CPAG, 2020).

### ‘Emergency’ food provision

COVID-19 has exposed the fragility of the food banking system. Food banks and other food aid providers are already at capacity and will struggle to adopt any additional responsibility required of them by central and/or local government. We recommend that the English administration follow the Scottish government’s cash-first approach and significantly increase hardship funds, administered by local authorities.

### Food Retail (Supermarkets and out-of-home)

Supermarkets should continue to monitor demand versus restrictions, whilst considering the needs and shopping practices of low income households. In cases where stocks are at sufficient levels, food banks should be allowed to buy volumes above the restricted quantities. Recognising the urgent need to re-establish food supplies to food banks amidst escalating demand, we recognise that, in the short-term, supermarkets may need to continue to support food banks with bulk food donations. However, in the medium- and long-term we encourage supermarkets to add their voices and influence to calls for government to accept responsibility for ending food insecurity. The response from the out-of-home sector has led to a range of new alliances to ensure food is reaching individuals who are required to self-isolate due to ‘vulnerability’. What can we learn from these initiatives going forward would be a useful area of further research.

## Conclusions

The government’s response to the economic crisis associated with COVID-19 has been impressive. The “self-employed income-support scheme” completes an unprecedented response package for workers, alongside the earlier “job retention scheme” for employees. Together, the two schemes protect up to 80% of individual average earnings or profits for those eligible for up to three months, and at a cost of many tens of billions. The £20 a week (£1,000 a year) increase in the value of Universal Credit, taking it to its highest real-terms value ever, after two decades of stagnation or declining value, will boost the incomes of millions of households. These policy changes have shown what is possible, and have underscored a key principle: ultimately, it is the government’s responsibility to protect population health, to guarantee household incomes, and to safeguard the economy. Millions of households were in poverty before the pandemic, and millions more will be so unless the government continues to protect household incomes through additional policy change. It is well-established that food banks - whether independent or affiliated to the Trussell Trust - and other food aid providers are unable to improve household incomes and mitigate food insecurity in the long-run. At present, they are fighting on all fronts but, ultimately, struggling in the face of this health and economic crisis.

## Data availability

No data are associated with this article.
