# Supplementary material for: How COVID-19 has exposed inequalities in the UK food system: The case of UK food and poverty
Source: Emerald Open Res. 2020 May 13;2:11. doi: 10.35241/emeraldopenres.13539.2 (PMC7219559; doi:10.35241/emeraldopenres.13539.2)
Supplement: Peer review history

# Peer review: Reviewer response for version 2

**DOI:** 10.21956/emeraldopenres.14775.r26810

**Published:** 2020-05-15

**Authors:** Peter Jackson

**Affiliations:** 1 Department of Geography, University of Sheffield, Sheffield, UK

The revised paper has addressed all of my previous concerns. Specifically, the authors have acknowledged the incomplete and inconsistent nature of the available evidence (inevitable when studying a current and rapidly-developing issue such as Covid-19). Their recommendations on reforming Universal Credit are more nuanced and the complexities associated with the term 'vulnerability' have been more fully developed.

Is the topic of the opinion article discussed accurately in the context of the current literature?

Yes

Are arguments sufficiently supported by evidence from the published literature?

Partly

Are all factual statements correct and adequately supported by citations?

Yes

Are the conclusions drawn balanced and justified on the basis of the presented arguments?

Yes

Reviewer Expertise:

Food security and sustainability particularly in relation to consumption issues

I confirm that I have read this submission and believe that I have an appropriate level of expertise to confirm that it is of an acceptable scientific standard.

# Peer review: Reviewer response for version 2

**DOI:** 10.21956/emeraldopenres.14775.r26807

**Published:** 2020-05-14

**Authors:** Rachel Loopstra

**Affiliations:** 1 Department of Nutritional Sciences, Faculty of Life Sciences and Medicine, King's College London, London, UK

Again, the authors should be congratulated for how quickly they wrote this important article. The paper highlights critical issues around vulnerability to food insecurity and of the food aid sector, which unfortunately, are only worsening as the COVID-19 crisis continues in Britain. 

 I appreciate the amendments that have been made in light of the feedback that was offered and have no further comments.

Is the topic of the opinion article discussed accurately in the context of the current literature?

Yes

Are arguments sufficiently supported by evidence from the published literature?

Partly

Are all factual statements correct and adequately supported by citations?

Partly

Are the conclusions drawn balanced and justified on the basis of the presented arguments?

Partly

Reviewer Expertise:

NA

I confirm that I have read this submission and believe that I have an appropriate level of expertise to confirm that it is of an acceptable scientific standard.

# Peer review: Reviewer response for version 1

**DOI:** 10.21956/emeraldopenres.14610.r26711

**Published:** 2020-04-20

**Authors:** Andrew Williams

**Affiliations:** 1 School of Geography and Planning, Cardiff University, Cardiff, UK

Overall, this is a well written paper which documents the immediate challenges facing, and inequalities underpinning, the UK food system in the early stages of the COVID19 pandemic. Writing as the events unfold is valuable for future research as it provides a critical snapshot at the start of the pandemic. To this end, the paper will be a useful reference point as the assortment of government, business, and civil society responses evolve over time.

 I have a few suggestions which the authors may wish to consider in their revisions of the article.

 Firstly, the role of austerity is a major absence and it seems important to include this if we are to understand the uneven capacity of local government responses to COVID19 and food insecurity. The revised paper might also include a critical discussion of government food aid for people who are deemed ‘clinically vulnerable’ and need to be shielded. The nutritional quality and contents of the food parcels are considerably variable across local authorities. Yet most of these parcels seem to mirror the tinned and ambient items in a foodbank parcel (with a miserly portion of fresh veg and fruit). Why? There is also emerging evidence of local authorities adopting their own schemes such as cash payments to households eligible for free school meals rather than issuing supermarket vouchers (for example, Rhondda Cynon Taf weekly payment of £19.50).

 Secondly, the paper is good at highlighting challenges facing the UK food aid sector, but an updated paper could offer an updated commentary on some of the organisational ‘fixes’ which have been developed by government, business and civil society as a response to these challenges. At times the position of the article is rather ambiguous. It critiques the corporatisation of food charity (page 6) then states, ‘supermarkets have pledged food donations to both the Trussell Trust and FairShare networks and this should continue’ (page 8). Alongside tightening the argument, I would invite the authors to offer their reflections on the ways the ‘emergency’ led to a contestation of radical and pragmatic sensibilities. What might be the long-term implications of the current response to COVID19 on the food aid sector? (NB: spelling FareShare). 

    

 Thirdly, a more variegated account of the food bank response is needed. The paper states ‘40% of [independents foodbanks] which operate on an open-access basis’. This needs a citation (Loopstra et al 2019) and a caveat about assessments and restrictions made by independent foodbanks to avoid a view that 40% independent foodbanks operate unlimited and direct access. The paper claims that referral agencies to foodbanks ‘have proved unviable in the context of increased demand, alongside the closure or partial shut down of many referral agents.’ Later the paper acknowledges how referral systems have increasingly utilised electronic / telephone referral. Perhaps bring these sections together.

 I found the discussion of how foodbanks have changed the way they work in response to COVID19 to be a little totalising. Following the transition period in the last month, some Trussell Trust distribution centres have informally adopted direct access, while some independents have adopted referral systems for the first time. Including the ways in which volunteers and distribution centres are doing things differently is important for the section on political and ethical debates on food charity. For example, the paper did not discuss how food parcels made by foodbank volunteers are now distributed to social services and council hubs to bypass the need for vouchers and ease the physical and bureaucratic burden on individuals to travel to the foodbank.  

 On page 7 the paper states that ‘instead of being invited into a building, service users are given a pre-packed food bank parcel, often at the threshold or delivered directly to their home (Trussell Trust, 2020c).’ From my experience this is not the case in every foodbank. This is an example where the tone of discussion needs to reflect the ambiguity and heterogeneity of how foodbanks have responded. Stating that ‘Care, sign-posting, and choice is removed from the interaction’ (page 7) is another example where I felt the claim is overstated. Although this is anecdotal evidence, people have come a Cardiff foodbank distribution centre requesting help with ringing utility companies, social services, and organising longer-term support from the council for those eligible. The volunteers who had experience in doing this were able to advise/signpost/ring on their behalf (if requested) while keeping social distance. Some food banks and volunteers are trying to find ways of ensuring dignity in the way they communicate social distancing rules and by offering choice even in the pre-made food parcels. Equally, the additional ‘policing’ of the movement of people (where to stand, sit, hand over the voucher) can be ‘doubly stigmatising’. Such rules, if conveyed insensitively, are likely to exacerbate feelings of shame for people who are using the foodbank for the first time. While this is highly contingent on layout and volunteers in each distribution centre, the experience is far more ambivalent than suggested here. Even so, this underlines the importance for the policy recommendations made in the paper (e.g. cash payments, cutting the 5 week for Universal Credit, increase Child Benefit).

 Fourthly, there are several points where there is opportunity to provide detailed evidence and citation. These sources were not available at time of publication but provide additional evidence on the challenges of COVID19 on the food system and household food insecurity, and responses to it:

- FAO (2020) Urban food systems and COVID-19: The role of cities and local governments in responding to the emergency ^1^.
- Loopstra R (2020) Vulnerability to food insecurity since the COVID-19 lockdown ^2^. 
- Lang T (2020) Feeding Britain. Penguin ^3^.

The critique of foodbank statistics as partial indication of household food insecurity is well established. A brief acknowledgement of this would suffice and would make space for the COVID discussion. The paper cites Cipriani (2020) as evidence for sharp increases in demand for food in foodbanks. Since publication of this paper, is there additional evidence to give a sense of the scale and significance of increased food bank usage?

 Alongside the discussion of panic buying and stockpiling, the authors might want to include how the just-in-time food system was disrupted by ‘less spectacular’ practices such as changing food consumption habits (‘eating in’) and shopping habits (weekly shop).

 In discussing ‘the empirical and ethical implications of Covid-19 for socio-economic inequalities in access to food in the UK’ the authors could be more explicit with the evidence on gender and race inequalities (see Women’s Budget Group (2020) Crises Collides: Women and Covid-19: Examining gender and other equality issues during the Coronavirus outbreak).

 On page 4 could you provide updated evidence for the ‘increased demands placed on the application system’ for Universal Credit? Also can you substantiate the claim made on page 5: ‘These volunteers tend to be older (over 70) and some have underlying health conditions; social distancing rules have caused many of these volunteers to stay away from food banks, placing considerable additional pressure on volunteers and staff continuing to distribute food.’

 Can the authors specify the contribution being ‘academic-practitioners’ makes to the paper? It is fairly common for academics especially in this field to have experience as frontline practitioners, policy and advocacy experience. What did the academic-practitioner duality bring to the type of evidence and analysis presented?

 Finally, connection between available data and the recommendations. One of the recommendations is for ‘hardship funds administered by local authorities, although these funds would need to increase substantially for this to be viable’. While it is not necessary for this paper, it would be useful to include a discussion of the uneven geography of hardship payments made across the UK (evidence of dwindling resources, gatekeeping and reliance on foodbanks). Another recommendation ‘that supermarket employees are paid the Real Living Wage, which will increase household income’ needs a detailed overview of pay and work conditions in UK supermarkets. More justification is needed of the proposed recommendations, not so much an assessment of alternatives but a defense against likely critiques of each policy intervention.

 Finally, I would like to thank the authors for writing a stimulating paper and to emphasise these comments are suggestions rather than a list of necessary revisions.

Is the topic of the opinion article discussed accurately in the context of the current literature?

Yes

Are arguments sufficiently supported by evidence from the published literature?

Partly

Are all factual statements correct and adequately supported by citations?

Partly

Are the conclusions drawn balanced and justified on the basis of the presented arguments?

Partly

Reviewer Expertise:

Geographies of welfare, food banks and poverty. Austerity and neoliberal governance.

I confirm that I have read this submission and believe that I have an appropriate level of expertise to confirm that it is of an acceptable scientific standard.

## References

[1] Urban food systems and COVID-19: The role of cities and local governments in responding to the emergency. *Food and Agriculture organisation of the United Nations*.2020; Reference source

[2] : Vulnerability to food insecurity since the COVID-19 lockdown. *The Food Foundation*.2020; Reference source

[3] : Feeding Britain.2020; Reference source

# Peer review: Reviewer response for version 1

**DOI:** 10.21956/emeraldopenres.14610.r26709

**Published:** 2020-04-17

**Authors:** Diane Holt

**Affiliations:** 1 University of Leeds, Leeds, UK

The start of this paper presents a useful summary of the (current) emergency measures put into place for income loss related to Covid-19 and an introduction to some of the issues facing the UK food supply system from the perspective of the most vulnerable in our society; those reliant of food banks and/or on lower incomes.

 Its strength lies in the authors’ focus on those in the UK relying on the food bank system and the exacerbation of their vulnerabilities (and those who will need to now access these resources) in this Covid-19 crisis. This nuanced exploration considers differences between those independent food banks and those corporate sponsors and presents a useful and timely overview of their emerging challenges. The blend of practitioners and academics who authored this paper allow us to see some of the aspects of the food bank challenges that might be hidden from view and that I hadn’t considered previously – such as the difficulties with volunteers who tend to be older and isolating. The paper provides an insightful overview of the different food bank types and the partnerships underpinning them. The recommendations presented focus on some of the initiatives that might support the clients of these food banks

 Recognising this is a paper is written in response to an ongoing and shifting crisis it thus provides a useful and timely introduction for readers. Where I would have liked to see more was on some of the more localised responses that seem to be emerging that are driven not by food banks themselves but by other routes to get food to vulnerable households. Good examples are some of the ad hoc initiatives emerging at a micro-local level for instance in schools who are delivering food to households of children with free school meals, and the role of other local actors such as local food retailers rather than the big supermarkets.

 There is also, perhaps not unsurprisingly, little recognition, of some of the supply issues impacting local producers who might need new payments terms, or are struggling themselves due to supply chain disruptions at second and third tier and those (like those supplying flowers) who have had their contracts cancelled as the product ranges narrow in stores in order to address the increased demand for staple products. There are also interesting shifts in some business models of food distributors who, unable to supply to hotels and restaurants, are shifting into a direct consumer market away from wholesale. They may have a role to play in reaching the most vulnerable in communities given their localised focus.

 Having spent time working within producer communities in sub-Saharan Africa and seen the global food supply chain ‘in action’ I recognise the vulnerabilities of this system and the communities that grow some of our imports in the developing world. This is mentioned but not developed further in this paper.

 The section on supermarket and retailer responses also seems a little underdeveloped. I know for instance that Sainsburys (as others) are initiating processes to deliver to vulnerable households. There are rapid innovations occurring in the UK food supply system that are happening in real time that may date this paper.

 Thus I see this as a timely (and of its time) interesting read. Living through food scarcities, supermarket queues, and trying to order for vulnerable family members the UK food system is very much on my mind at present. Therefore, I do see this as a useful and valuable addition to our knowledge base and a paper that provide some insightful contributions. I would like to see in future work a more longitudinal focus that tracks what has happened and the aftermath

Is the topic of the opinion article discussed accurately in the context of the current literature?

Yes

Are arguments sufficiently supported by evidence from the published literature?

Yes

Are all factual statements correct and adequately supported by citations?

Yes

Are the conclusions drawn balanced and justified on the basis of the presented arguments?

Yes

Reviewer Expertise:

Informal economy supply chains, social enterprise, green supply chains, producer communities, sustainable development

I confirm that I have read this submission and believe that I have an appropriate level of expertise to confirm that it is of an acceptable scientific standard.

# Peer review: Reviewer response for version 1

**DOI:** 10.21956/emeraldopenres.14610.r26712

**Published:** 2020-04-14

**Authors:** Alex Murdock

**Affiliations:** 1 London South Bank University, London, UK

The article is about a fast moving situation and naturally reflects the situation at the time of writing.  The academic sources are all fine and appropriate but I would gently suggest some reconsideration as to whether to include reference to the following (which would not be found in 'academic ' literature but rather in contemporary 'grey' literature accounts):

1. The challenge of self isolation of the 'older and vulnerable' represents a major challenge to food banks in terms of their volunteer demographic. This has featured in recent accounts and is reflected (online) by the number of Trussell trust food banks shown as closed; It is a moving target though which may have been partly addressed by new and less vulnerable volunteers... It is NOT just about the food.
2. There is significant evidence of emerging partnerships and innovative responses to food issues in COVID - many of these are local ( Food Bikers in Cornwall for example) - Crisis sometimes generates solutions.
3. It terms of UK food insecurity the perceived threat of no migrant workers to gather crops may be resolved by an influx of domestic workers - some laid off by COVID (see the HOPS site which is no longer accepting applications until May due to an apparent surplus of offered labour. However this is a 'moving target' - see Metro link below ^1^

Factually the piece is well written and paints a good picture with strong analysis... I only query whether it misses some of the more recent developments. For example Morrisons have reportedly given £10M to the food banks...possibly one of the largest single money donations in UK food bank history?? This was a direct result of COVID - it is over  £20K per Trussell trust food bank by my rough estimate? ^2^.

Is the topic of the opinion article discussed accurately in the context of the current literature?

Partly

Are arguments sufficiently supported by evidence from the published literature?

Yes

Are all factual statements correct and adequately supported by citations?

Yes

Are the conclusions drawn balanced and justified on the basis of the presented arguments?

Yes

Reviewer Expertise:

Food Bank and Food Insecurity, Social Innovation,  Active Ageing

I confirm that I have read this submission and believe that I have an appropriate level of expertise to confirm that it is of an acceptable scientific standard.

## References

[1] : UK facing food shortages unless more people take jobs on farms. Reference source

[2] : Morrisons gives food banks £10m during coronavirus outbreak. Reference source

# Peer review: Reviewer response for version 1

**DOI:** 10.21956/emeraldopenres.14610.r26710

**Published:** 2020-04-07

**Authors:** Rachel Loopstra

**Affiliations:** 1 Department of Nutritional Sciences, Faculty of Life Sciences and Medicine, King's College London, London, UK

It is excellent to see to this reflective and timely piece on the impacts of the COVID-19 crisis on food insecurity, food aid systems, and food systems more broadly. The authors are well placed to offer their perspectives and comments on these events and outline their reasons for doing so now, at this moment (or the moment one week ago), to chronicle events as they happen. As the situation evolves, new commentaries will be appropriate, but to observe and comment on multi-faceted repercussions of the COVID-10 as they happen is also important as they have the opportunity to shape the response in the future, potentially even in the very short term. Important issues are discussed throughout the article, and juxtaposing vulnerability in food supply systems alongside issues in food banks and how the two are connected is useful.

 Below I offer some recommendations for how points could be strengthened by making reference to evidence and sources, but also pointing out where evidence is scant and anecdotal; a critical part of this story is that food banks and other forms of food aid are largely left to operate without scrutiny or evidence of their actual ability to ensure that people receive food when they are faced with food insecurity.

 Key weaknesses in the operations of food banks exposed by the COVID-19 crisis are discussed, but it might be useful to point out the research that has documented and discussed different elements of fragility in the system before this crisis. It has been a long-standing concern among researchers that food charity cannot and should not be relied upon to provide food to people at risk of food insecurity. This is because the system itself cannot provide “security” given the system is so contingent on voluntary donations and volunteer labour. Instances of food banks running low on food and at times, having to cut down on the size of food parcels or turn people away have been documented before. It would also be useful to more explicitly outline what food bank closures and limited food supplies will mean for people who have come seeking help from food banks: what might it mean for someone who is facing not having enough food to then not be able to access their local food bank? Some references to incorporate on documented weaknesses in food bank systems:

- Tarasuk, V., Dachner, N., Hamelin, A. et al. A survey of food bank operations in five Canadian cities. BMC Public Health 14, 1234 (2014) ^1^.
- Loopstra, R., Lambie-Mumford, H. & Fledderjohann, J. Food bank operational characteristics and rates of food bank use across Britain. BMC Public Health 19, 561 (2019) ^2^.

Some caution is needed in reporting on the effects of Covid-19. It would be useful to see caveats such as “media reports from talking to frontline food bank managers and volunteers found…” with reference to comments about the increase in food bank demand and the reasons for it. These observations from individual food banks may bear out in data from food banks, but without systematic collection and reporting on this, it is important to note that this is anecdotal evidence, likely from food banks experiencing the greatest increases in demand.

 With reference to the Government’s CJRS, it might be useful to also highlight that 80% of wages will be paid up to £2,500 before tax. This means people earning over £30,000 will have relatively higher reductions. While it is possible that they are a less economically vulnerable group, this is highly dependent on how many dependents they have in their household and where they live. They may also have higher fixed costs, such as mortgage/rent payments, house insurance etc.

 Comments on “Emerging fragilities within the UK food aid system” could be better supported by references and more description. For those not familiar with FareShare, it would be useful to describe how they operate and to include reference to their website. In terms of how food banks work, you could include a reference to the Trussell Trust’s website and also to our survey of independent food banks in England, which documents sources of food donations ( https://www.foodaidnetwork.org.uk/independent-food-bank-survey).

 It is good to highlight the level of debt that the poorest have, but also their lack of savings to withstand financial shocks. The ONS also reports some statistics on this here: https://www.ons.gov.uk/peoplepopulationandcommunity/healthandsocialcare/conditionsanddiseases/articles/coronaviruscovid19roundup/2020-03-26.

 The variation between the extent of food bank use and numbers experiencing food insecurity are also sharply revealed by the estimates of number of people who are food insecure and estimated number of individuals who use food banks. See for example Loopstra et al. (2019) ^3^ where estimated number of adults using Trussell Trust food banks was 321,599 adults in 2016-17 and estimated number food insecure based on the Food and You 2016 data was 10.2 million, including marginal food insecurity. Even if food bank use figures were doubled to account for independent food bank use, there is a wide discrepancy. The more recent State of Hunger report also provides an estimate of the discrepancy between food insecurity and food bank use figures.

- Loopstra R, Reeves A, Tarasuk VThe rise of hunger among low-income households: an analysis of the risks of food insecurity between 2004 and 2016 in a population-based study of UK adultsJ Epidemiol Community Health 2019;73:668-673 ^3^.
- https://www.trusselltrust.org/state-of-hunger/

Reference is made to a survey of households conducted in York – “20% of households reporting food insecurity had ever used a food bank”, but as this survey was conducted among households with children in primary school, a more refined description of this survey should be provided. Single individuals, particularly men, are an important demographic in food banks, thus, the discrepancy between food bank use and food insecurity among households with children may not be the same for other household demographics.

 When discussing the distribution of food insecurity in the population, it might be useful to reference our multi-variate analysis of the 2016 Food and You data (Loopstra et al. 2019, *ibid*). It highlights which socio-economic characteristics are independently associated with higher odds of food insecurity, but also the characteristics associated with severe food insecurity in particular. Very relevant to note here is how people with health conditions, and particularly those for whom their conditions limit their daily activities (i.e. those by definition with a disability) are at higher risk of food insecurity.

 The meaning of this statement is not clear to me: “Sustained and, particularly, hidden food insecurity is, thus, both an individual and a societal issue.” First, I would argue that no one should ever have to go without food, so whether sustained or a single episode, food insecurity is a societal issue. Second, hidden food insecurity is certainly important, but in the context of the scale of the problem now being tracked beyond food bank use statistics through the Food and You survey and monitoring in Scotland, as well as forthcoming monitoring through the Family Resources Survey, it isn’t clear what hidden food insecurity refers to. Is this among people not captured in household surveys (importantly here, people who are homeless and people who are institutionalised) or among people who do not respond to household surveys, or among people who do not give honest responses?

 The discussion of the impacts of school closures on access to food among Free School Meal recipients is useful but focuses on the experiences of children who are eligible to receive them or who may newly become eligible to receive them on account of unemployment or reductions in income. But here, it would be useful to highlight concerns about eligibility: how low the income threshold is for working families (for example, see https://cpag.org.uk/welfare-rights/resources/article/universal-credit-and-free-school-meals) and families with no recourse to public funds who have newly become unemployed who are not eligible.

 With reference to growing vulnerability on account of self-isolation rules, distinguishing between vulnerability to food insecurity arising from a lack of finances from that arising from a lack of physical access to food would be useful here. Food banks focus on reaching people affected by the former. For example, on the Trussell Trust website, this statement can be found: “If you can afford food or aren’t in financial difficulty but can’t get to the shops because you’re self-isolating, you should contact your local council or Citizens Advice for support. Food banks can only help people who don’t have enough money for food and many are having to run reduced services in order to do this safely during the outbreak, so they’re not best placed to deliver to you.”

 ( https://www.trusselltrust.org/coronavirus-food-banks/faqs/#1585815322735-9695c857-a1d6) Discussion of local government and central government responses- and their limitations (i.e. only a fraction of people at risk of severe illness from coronavirus on account of an underlying specific health condition have been identified by government) would be useful. Whilst schemes suggest the government is showing responsibility for vulnerability arising from health issues, they are likely not going far enough, and they are relying (once again) on charities to fill the gap, even though it is clear that some charities, as in The Trussell Trust’s statement, do not feel it appropriate for them to be charged with this task.

 A question is raised: “can a clear distinction be made between individuals seeking food support due to income and those requesting assistance due to health?” But clearly a distinction can be made, even though many people who do not have sufficient incomes also have health conditions. If someone has money in the bank, but cannot use these finances to access food because they cannot get to shops or get a food delivery, this is a problem of physical access. Others have no money in the bank and also cannot go out to shops or get a food delivery. The former group also likely do not have the same array of other disadvantages as the latter such as lack of social network, lack of money for phones or internet, risk of not being able to make housing payments and other bills, etc. given how people in poverty experience multiple disadvantages.

 Local Government Resilience Forums are mentioned, but not defined. As above, it would be useful to see discussion on how governments are responding to food access for those being told to self-isolate for 12 weeks, as this highlights where responsibility is being taken and reveals gaps where people are being left without support.

 The last paragraph on page 6 needs reference to sources. What is the reference for “40% of independent food banks operate on an open-access basis”? and what is the source of evidence that independent food banks are receiving clients who have been unable to receive a referral to a Trussell Trust food bank? (and is this a new phenomenon or could this not always be the case?)

 When referencing the study of independent food banks (Loopstra R, Goodwin S, Goldberg B, et al.: A survey of food banks operating independently of The Trussell Trust food bank network. 2019.), it is important to point out that this was only a survey of independent food banks operating in England.

 The recommendations, whilst all very important, do not necessarily arise from the evidence reviewed in the body of the paper. For example, more could have been said about the demographic of people who use food banks and who will now also be left insufficiently protected by government assistance. This discussion necessitates discussion of the rates of welfare benefits in the body of the paper. The recently announced increase in universal cred it also begs a question about whether people who currently are using food banks because universal credit is insufficient to cover their basic needs will now be at lower risk of food insecurity.

 Another important issue is the lack of financial resilience built into the social security system. The barely enough (and often not enough) levels of assistance (emulated by calculations of Minimum Income Standards: https://www.jrf.org.uk/report/minimum-income-standard-uk-2019) mean that many people living on these incomes cannot save and therefore have no financial buffer when exposed to shocks.

 Discussion of wages of supermarket staff are important, but it is not evidenced in the paper that they are not paid the Real Living Wage already. What proportion of supermarkets do not pay this?

 It is not clear why supermarkets would he charged with implementing a public health initiative akin to Healthy Start. Why would this not be initiated by government and available across supermarkets?

 The recommendation that supermarkets continue to pledge food donations to food charities seems out of line with discussion in the paper about concerns about the corporatisation of food and reliance on food aid systems in general. Again, it is not clear why supermarkets are being charged with this.

 Under recommendations for “Emergency Food Provision”, it is recommended that cash grants be provided. Who these should be provided to at this time is not explained. How these would work for people facing self-isolation is also not clear. This section would be enhanced by delineating between recommendations to prevent food insecurity arising from the financial shocks caused by the crisis and the food insecurity arising from a lack of physical access to food. Reiterating why it is important that no one must rely on food banks at this time would be useful.

Is the topic of the opinion article discussed accurately in the context of the current literature?

Yes

Are arguments sufficiently supported by evidence from the published literature?

Partly

Are all factual statements correct and adequately supported by citations?

Partly

Are the conclusions drawn balanced and justified on the basis of the presented arguments?

Partly

Reviewer Expertise:

NA

I confirm that I have read this submission and believe that I have an appropriate level of expertise to confirm that it is of an acceptable scientific standard, however I have significant reservations, as outlined above.

## References

[1] : A survey of food bank operations in five Canadian cities. *BMC Public Health*.2014;14: 10.1186/1471-2458-14-1234 1234 10.1186/1471-2458-14-1234 25432209PMC4289166

[2] : Food bank operational characteristics and rates of food bank use across Britain. *BMC Public Health*.2019;19(1) : 10.1186/s12889-019-6951-6 561 10.1186/s12889-019-6951-6 31088456PMC6518699

[3] : The rise of hunger among low-income households: an analysis of the risks of food insecurity between 2004 and 2016 in a population-based study of UK adults. *J Epidemiol Community Health*.2019;73(7) : 10.1136/jech-2018-211194 668-673 10.1136/jech-2018-211194 31036607

# Peer review: Reviewer response for version 1

**DOI:** 10.21956/emeraldopenres.14610.r26713

**Published:** 2020-04-06

**Authors:** Peter Jackson

**Affiliations:** 1 Department of Geography, University of Sheffield, Sheffield, UK

This is an interesting and timely paper that reviews the way the current Coronavirus pandemic (COVID-19) has drawn attention to, and exacerbated, inequalities in the UK food system. The paper has a particular focus on those who rely on foodbanks and other forms of ‘emergency’ food aid. It raises interesting questions about the way social scientists can respond to ongoing crises, where reliable data are not readily available. This is particularly striking in relation to the evidence on which the authors base their Recommendations where the data is, necessarily, incomplete and of inconsistent quality.

 The authors outline the way COVID-19 has drawn attention to the fragility of the food supply chain. This has been both short-term (in response to panic buying) and longer-term (in relation to our reliance on imported food). A useful further reference here would have been Tim Lang’s recent book on Feeding Britain (2020).

 The authors claim to have a ‘unique perspective’ on these issues because of their combined experience of food policy, research and advocacy. I thought this was somewhat overplayed and would be true of many research teams and co-authored papers. More specifically, they use their expertise ‘to chronicle and assess’ the available evidence (p.3). More could have been said about the fragmentary nature of the evidence and the challenges of assessing a situation that is changing on a daily basis.

 The paper was written within the first few weeks of the COVID-19 pandemic affecting the UK.  This gives it an urgency, reflecting on issues as they evolve in more-or-less ‘real time’. It also poses problems in terms of the evidence on which the paper is based including sources (newspapers, grey literature) which do not meet the highest (peer reviewed) standards of social science evidence. Similarly, phrases like ‘in recent days’ are unhelpful in a rapidly evolving context and would be better replaced with a specific date.

 While the authors provide a good review of the challenges facing people on low-incomes, especially those requiring access to Universal Credit, more could have been said about the way current policies such as social distancing and self-isolation have exacerbated problems of accessing safe, nutritious food.

 The question of ‘vulnerability’ could also have received more attention. It wasn’t clear whether or not the authors subscribe to the conventional view that people are vulnerable because of their demographic or medical status (as elderly, pregnant or with an underlying condition). This definition has been challenged (in the context of foodborne disease) where an alternative definition seems preferable, based on a ‘pathways and practices’ approach. Rather than seeing all elderly people as equally at risk, for example, this approach focuses on the pathways and practices that increase their vulnerability.  I wondered if this approach (outlined in Critical Public Health, 2018) might also be relevant here?

 The most problematic part of the paper, in my view, is the way the Recommendations are underpinned by appropriate evidence. While I would personally support supermarket workers being paid a Real Living Wage or the abolition of the 5-week wait for Universal Credit or price promotions on fruit and vegetables, one would (in other circumstances) expect to see a more robust review of the pros and cons of these policies. Would there be a sufficient supply of fruit and veg to meet the likely increased demand? What bureaucratic and other challenges would need to be addressed by an immediate switch in eligibility rules? These are just examples of the problematic link between evidence and policy prescription that arise in ‘emergency’ conditions such as we are currently facing and the authors may argue that it is preferable to base policy shifts on incomplete evidence if the alternative is worse in terms of its adverse effects on those already most ‘vulnerable’. But these issues demand further exploration in my view.

 Finally, there are some minor editorial issues that should be addressed in any future revisions of the paper: ‘mitigate against’ is ungrammatical (not to be confused with ‘militates against’).  The phrase is used repeatedly throughout the paper and should simply be ‘mitigate’.  The References are not in correct alphabetical order, and the phrase ‘pregnant mothers’ (page 8) should be ‘pregnant women’.

Is the topic of the opinion article discussed accurately in the context of the current literature?

Yes

Are arguments sufficiently supported by evidence from the published literature?

Partly

Are all factual statements correct and adequately supported by citations?

Yes

Are the conclusions drawn balanced and justified on the basis of the presented arguments?

Yes

Reviewer Expertise:

Food security and sustainability particularly in relation to consumption issues

I confirm that I have read this submission and believe that I have an appropriate level of expertise to confirm that it is of an acceptable scientific standard, however I have significant reservations, as outlined above.
